# Endovascular treatment of thoracoabdominal aortic aneurysm: a case report

**DOI:** 10.1186/1752-1947-4-37

**Published:** 2010-02-02

**Authors:** Arash Mohammadi Tofigh, Massoud Ghasemi, Babak Heidari Aghdam, Mersedeh Karvandi, Afsoon Kaboli

**Affiliations:** 1 Imam Hossein Medical Center, Shahid Beheshti University of Medical Sciences, Tehran, Iran; 2 Research Center of Endovascular Intervention, Tehran, Iran; 3 Taleghani Hospital, Shahid Beheshti University of Medical Sciences, Tehran, Iran

## Abstract

**Introduction:**

Thoracoabdominal aortic aneurysms usually present in elderly patients with serious renal, pulmonary, cerebral, or cardiac comorbidities that pose a great challenge to the attending surgeon. Endovascular techniques for the treatment of thoracoabdominal aneurysms are not yet widely used due to limitations associated with them, such as spinal and visceral ischemia.

**Case presentation:**

An 87-year-old Caucasian man with a symptomatic Crawford type I thoracoabdominal aortic aneurysm was treated successfully with a long tube stent graft using endovascular techniques and without any complication in follow-up examinations. The stent was placed distal to the left subclavian artery, and proximal to the celiac axis.

**Conclusion:**

The use of endovascular stents for long segment thoracoabdominal aortic aneurysms needs to undergo clinical investigation to determine whether this procedure decreases morbidity and mortality rates.

## Introduction

Advancements in diagnostic techniques have made the detection of thoracoabdominal aortic aneurysm feasible, with most centers reporting an increase of 5% in the detection of suprarenal aneurysms. Thoracoabdominal aortic aneurysms are usually identified in elderly patients with serious renal, pulmonary, cerebral, or cardiac comorbidities that pose a great challenge to the attending surgeon [1]. Surgery necessitates a thoracoabdominal incision that will approach the aneurysm through the retroperitoneum and mobilize the visceral organs medially. In some cases, the surgeon utilizes cardiopulmonary bypass to perfuse the distal vessels, hoping to decrease the incidence of paraplegia [2].

The advent of endovascular aortic prosthesis provides patients with alternative therapy which hopes to decrease the morbidity and mortality of surgery [3]. Endovascular techniques are well described for abdominal and thoracic aortic aneurysms. These techniques are quite new as applied to thoracoabdominal aneurysms, however, due to serious adverse events such as spinal and visceral ischemia following the procedure [4]. We describe in this case report a patient with an extensive Crawford type I thoracoabdominal aneurysm treated with the placement of a stent graft in the thoracic aorta using endovascular techniques.

## Case presentation

**Figure 1:**
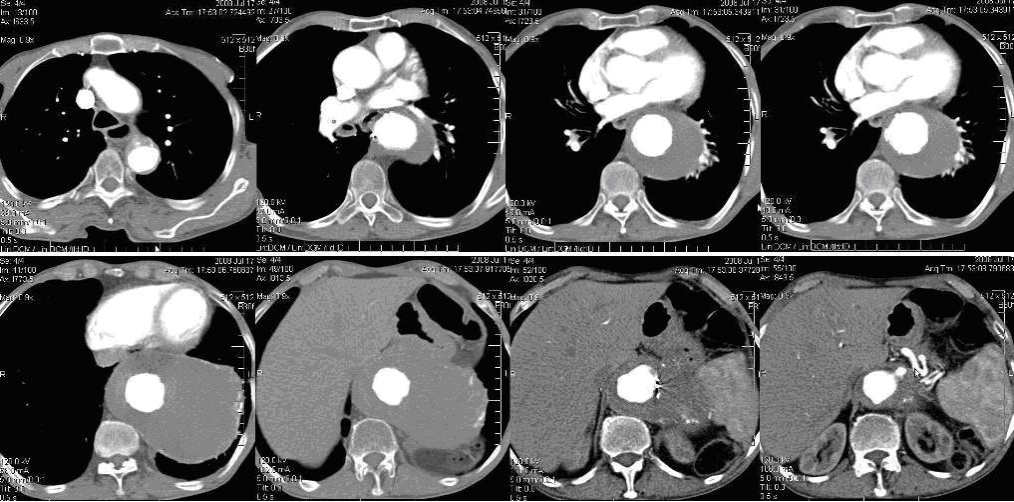
**Computed tomography scan showing the patient's Crawford Type I thoracoabdominal aneurysm**.

An 87-year-old Caucasian man presented to our institution with chest and epigastric pain radiating to his back. Computed tomographic scans were performed urgently, and these showed a large Crawford type I thoracoabdominal aortic aneurysm (Figure 1). The aneurysm measured 17 cm in length and was 5 cm to 6 cm distal from the left subclavian artery and 2 cm to 3 cm proximal to the celiac axis. The maximum anteroposterior diameter of the aneurysm was 13 cm above the diaphragm. The aorta between the celiac axis and the renal arteries was of normal size. Another aneurysm measuring 6 cm in length and 4 cm in diameter with no extension to the iliac arteries was detected distal to the renal arteries.

Our patient was not in a fit condition to undergo open surgery, so endovascular surgery was the preferred option. The diameters of the proximal and distal necks of the aneurysm were 3.2 cm and 3.4 cm, and a large mural thrombosis was present along all its length. We decided to treat our patient with a single tube stent graft. Although a long segment of the aorta would be covered during the procedure, we considered that there was a low chance of spinal ischemia, as the mural thrombosis had plugged all of his intercostal and lumbar arteries. Cerebrospinal fluid drainage was considered as a protective move for spinal circulation during the procedure.

**Figure 2:**
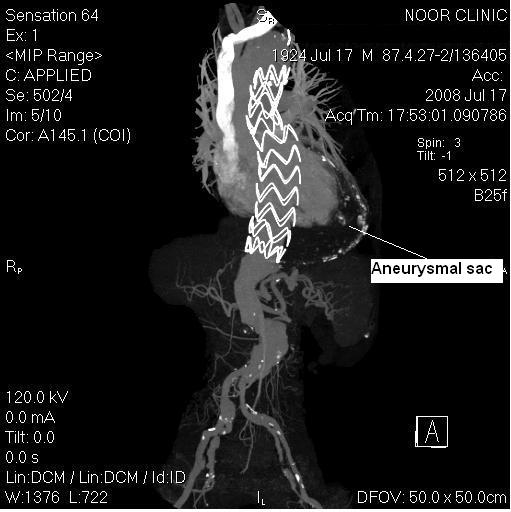
**Follow-up computed tomography angiography showing a total exclusion of the aneurysm by the stent graft**.

Under general anesthesia, our patient's right femoral artery was dissected and controlled. An endovascular stent graft (VALIANT TF 4242C200X, Medtronic) was deployed distal to the left subclavian artery, thus covering the aneurysm. The stent graft was 21 cm in length and was placed just above his celiac axis. We controlled the proximal end deployment by real-time transesophageal echocardiography, and the distal end deployment under angiography. Follow-up transesophageal echocardiography, computed tomography and angiography showed a complete exclusion of the thoracoabdominal aneurysm (Figure 2). Correction of the abdominal aortic aneurysm was programmed for later. The patient was discharged three days after the procedure and showed no complications during the succeeding nine months.

## Discussion

The surgical treatment of thoracoabdominal aneurysms poses a major challenge to the vascular surgeon, especially because patients are usually elderly and have serious comorbidities. The development of endovascular prostheses has greatly improved the treatment of patients with aneurysm or dissection in the thoracic and abdominal aorta [4-6]. This is a major achievement in the therapy of these types of aneurysms, but it necessitates precise diagnostic investigations to determine the proximal and distal necks of the aneurysm, the vessels involved in the aneurysm, and the location of tears in aortic dissection. One of the major challenges is the risk of paraplegia in patients who undergo surgical treatment of these aneurysms. Short cross-clamp time, distal perfusion, hypothermia, cerebrospinal fluid drainage and the use of steroids are some of the strategies aimed at decreasing the risks of surgery [7].

The use of endovascular stents could potentially decrease the risk of paraplegia and serious morbidities associated with the surgical approach. Using a long tube stent graft to exclude the aneurysm is a new approach to treat thoracoabdominal aortic aneurysms. However, serious complications like visceral and spinal ischemia should still be considered before surgery. Graft distortion will be considered as a potential risk in longer stent grafts and controlling the whole procedure using real-time angiography and transesophageal echocardiography is very important to avoid this complication [8]. Chuter *et al. *[9] developed a multi-branched stent graft for the treatment of thoracoabdominal aneurysm that would decrease the risk of visceral ischemia. This device, however, is still in the experimental phase.

## Conclusion

The use of endovascular stents for long segment thoracoabdominal aortic aneurysms will have to undergo clinical investigation to determine whether the stents decrease the morbidity or mortality rates associated with the condition. Although the patient described in this case report is doing well at nine months, a longer follow-up time is needed to further identify the beneficial effects of this new approach to a complex problem.

## Patient's perspective

Following the intervention our patient said that he had not believed that his condition would be treated so easily and he had expected a serious open surgery.

## Consent

Written informed consent was obtained from the patient for publication of this case report and any accompanying images. A copy of the written consent is available for review by the Editor-in-Chief of this journal.

## Competing interests

The authors declare that they have no competing interests.

## Authors' contributions

AMT served as the vascular surgeon to the patient described in this case report. MG, AK and BHA were the interventionists, while MK was the echocardiologist. All authors read and approved the final manuscript.
